# Identifying Predictors of Taxane-Induced Peripheral Neuropathy Using Mass Spectrometry-Based Proteomics Technology

**DOI:** 10.1371/journal.pone.0145816

**Published:** 2015-12-28

**Authors:** Emily I. Chen, Katherine D. Crew, Meghna Trivedi, Danielle Awad, Mathew Maurer, Kevin Kalinsky, Antonius Koller, Purvi Patel, Jenny Kim Kim, Dawn L. Hershman

**Affiliations:** 1 Department of Pharmacology, Columbia University Medical Center, New York, New York, United States of America; 2 Department of Medicine, Columbia University Medical Center, New York, New York, United States of America; 3 Department of Epidemiology, Columbia University Medical Center, New York, New York, United States of America; 4 Herbert Irving Comprehensive Cancer Center, Proteomics Shared Resource, Columbia University Medical Center, New York, New York, United States of America; Moffitt Cancer Center, UNITED STATES

## Abstract

Major advances in early detection and therapy have significantly increased the survival of breast cancer patients. Unfortunately, most cancer therapies are known to carry a substantial risk of adverse long-term treatment-related effects. Little is known about patient susceptibility to severe side effects after chemotherapy. Chemotherapy-induced peripheral neuropathy (CIPN) is a common side effect of taxanes. Recent advances in genome-wide genotyping and sequencing technologies have supported the discoveries of a number of pharmacogenetic markers that predict response to chemotherapy. However, effectively implementing these pharmacogenetic markers in the clinic remains a major challenge. On the other hand, recent advances in proteomic technologies incorporating mass spectrometry (MS) for biomarker discovery show great promise to provide clinically relevant protein biomarkers. In this study, we evaluated the association between protein content in serum exosomes and severity of CIPN. Women with early stage breast cancer receiving adjuvant taxane chemotherapy were assessed with the FACT-Ntx score and serum was collected before and after the taxane treatment. Based on the change in FACT-Ntx score from baseline to 12 month follow-up, we separated patients into two groups: those who had no change (Group 1, N = 9) and those who had a ≥20% worsening (Group 1, N = 8). MS-based proteomics technology was used to identify proteins present in serum exosomes to determine potential biomarkers. Mann–Whitney–Wilcoxon analysis was applied and maximum FDR was controlled at 20%. From the serum exosomes derived from this cohort, we identified over 700 proteins known to be in different subcellular locations and have different functions. Statistical analysis revealed a 12-protein signature that resulted in a distinct separation between baseline serum samples of both groups (q<0.2) suggesting that the baseline samples can predict subsequent neurotoxicity. These toxicity-associated biomarkers can be further validated in larger retrospective cohorts for their utility in identifying patients at high risk for CIPN.

## Introduction

Chemotherapy-induced peripheral neuropathy (CIPN) is a common and potentially disabling side effect of many anticancer drugs.[1] The symptoms are predominantly sensory and present in a “stocking-glove” distribution as pain, numbness, or tingling in the hands or feet.[2] The pathogenesis of CIPN is not well understood.[2, 3] In a recent meta-analysis of 31 CIPN studies involving 4179 patients on various neurotoxic chemotherapy agents, the aggregate prevalence of CIPN was 48%.[4] CIPN has important clinical implications in the treatment of cancer patients as it can result in dose reductions or discontinuation, which may ultimately affect overall survival.[2] Further, for cancer survivors, the CIPN symptoms can significantly impact quality of life.[1, 5, 6] The course of CIPN can be unpredictable and although some symptoms may improve with time, other symptoms may persist or worsen as a result of permanent nerve damage.[1] While there has been extensive research on agents for the prevention and treatment of CIPN, therapeutic options remain limited.[7]

There is interest in identifying factors that predict risk for developing CIPN, as this will allow physicians to personalize treatment choice. Several studies have used genome-wide association studies (GWAS) to identify genetic predictors of CIPN; however, the results have not been consistent. Single nucleotide polymorphisms (SNPs) in genes such as *FANCD2*[8], *FDG4*[9], *EPHA5*[9], and *CYP2C8*[10, 11] have been associated with CIPN. But the evaluation of CIPN and exposure to chemotherapy drugs has differed between studies.[2, 12] In addition, studies using GWAS have often been inadequately powered to make meaningful conclusions.[10] More importantly, disappointing results from a recent randomized double-blinded clinical trial for the prevention of taxane-induced neuropathy for breast cancer patients strongly suggest that the need for human specific and disease specific knowledge on chemotherapy-induced toxicity. With regard to assessment of CIPN, the majority of studies have used the Common Terminology Criteria Adverse Event (CTCAE) grading scale reported by health care providers, which is less sensitive and less reliable than patient reported outcome measures.[13, 14] Finally, these studies were unable to account for host factors that may affect the development of neuropathy, such as concurrent medications and underlying comorbidities such as diabetes.[12]

Recent advances in next generation sequencing technologies have supported the discoveries of a number of pharmacogenetic markers that predict response to chemotherapy.[15] However, effectively implementing these pharmacogenetic markers in the clinic remains a major challenge. On the other hand, recent advances in proteomic technologies incorporating mass spectrometry (MS) for biomarker discovery show great promise in providing comprehensive knowledge of molecular profiles from complex biological samples and implementing the identified protein biomarkers rapidly in the clinic. In this study, we tested the feasibility of applying a MS-based biomarker discovery workflow to identify biomarkers of taxane-induced peripheral neuropathy from proteins that are packaged and protected in exosome microvesicles in the serum. It has been reported that exosomes remain intact in biofluids during long-term storage, and therefore serve as an excellent reservoir for biomarker discovery. Also, it has become increasingly clear that exosomes have specialized function[16] instead of mere cell debris. Proteomic cataloguing of exosomes from diverse cell types has revealed a common set of membrane and cytosolic proteins, suggesting the evolutionary importance of these membrane particles.[16, 17] Together with results from our studies, we demonstrated that exosomes represent a new type of intercellular messenger.

## Methods and Materials

### Materials

High-performance liquid chromatography (HPLC) grade LC buffers, Dithiothreitol (DTT), acetonitrile (ACN), ammonium bicarbonate, trifluoroacetic acids (TFA), and iodoacetamide (IAA) were purchased from Thermo Fisher Scientific (Waltham, MA). Trypsin Gold, mass spectrometry grade, was purchased from Promega (Madison, WI). Nanopure water was prepared with use of Milli-Q water purification system (Millipore, Billerica, MA).

### Study cohort

We conducted a nested case-control study within a prospective cohort of 50 women with early stage breast cancer receiving adjuvant taxane-based chemotherapy. Patients were informed of the investigational nature of the study and signed informed consent. The study was conducted after appropriate approval by individual institutional review boards in compliance with the provisions of the Declaration of Helsinki and Good Clinical Practice guideline. The cohort study was approved by the Columbia University Institutional Review Board and all patients signed written informed consent. The patients were monitored for neuropathy using quantitative sensory testing, the Functional Assessment of Cancer Therapy-Neurotoxicity Subscale (FACT-Ntx) score, and a neuropathic pain scale every four weeks during active therapy and every three months for a total of one year thereafter. A physical examination and blood sample were conducted at these time points. Demographic and medical information were collected using self-report measures and via medical chart review. The details on the methods have been previously published.[13]

The FACT-Ntx subscale (score range 0–44) was calculated at each time point such that higher score indicates less neuropathy.^8^ CIPN was defined as a ≥ 20%[18] worsening in FACT-Ntx score (lower score) from baseline to any of the following time points: completion of taxane chemotherapy, 6 months, or 12 months after completion. Based on this definition, two groups were created: 1) patients with CIPN (Group 1); 2) patients with no change in their FACT-Ntx score (Group 2).

### Mass spectrometry (MS)-based proteomics technology

#### Isolation of total exosome from human serum

100μl of serum were used to purify total exosome using the manufacturer’s protocol with the following modifications. We used 40μl of the reagent in the kit for 100μl of serum and we washed the exosome pellet twice with PBS. Total exosome lysate was then generated in 50μl of the lysis buffer (50mM Ammonium Bicarbonate, 4M Urea, and protease cocktail) using 1.4mm ceramic beads and the Omni Bead Ruptor Homogenizer (Omni International, GA). Protein concentration in total exosome lysate was determined by the EZQ Protein Quantification Assay (Life Technology Corp. CT).

#### Immunoblotting for exosome enrichment

Total exosome lysate in the unbound (supernatant) and enriched fractions were electrophoresed in 10% SDS polyacrylamide gel, transferred to nitrocellulose membranes, and blocked with BSA. CD63 antibody (1:1000 dilution, H-193, Santa Cruz Biotechnology, Inc, TX) was used as an indication of exosome enrichment. Ponceau S staining was used to visualize equal loading of both fractions. Signals were detected using extended chemiluminescence.

#### Trypsin digestion for MS analysis

125ng of trypsin was added to 5μg of total exosome lysate (1:40) along with 2mM CaCl_2_ and incubated at 37°C for 16 hours. Samples were centrifuged subsequently for 30 minutes at 14,000 rpm, and the cleared supernatants were transferred to fresh tubes to be acidified with 90% formic acid (2% final) to stop proteolysis. The soluble peptide mixtures were collected and dried using Speedvac for LC-MS/MS analysis.

#### LC-MS/MS analysis

The concentrated peptide mix was reconstituted in a solution of 2% acetonitrile (ACN) and 2% formic acid (FA) for MS analysis. Peptides were loaded with the autosampler directly onto a 2cm C18 PepMap pre-column and were eluted from the 15cm x 75 μm ID PepMap RSLC C18, 3μm column using a Thermo Easy-nLC1000 UHPLC with a 70 min gradient from 2% buffer B to 30% buffer B (100% acetonitrile, 0.1% formic acid). The gradient was switched from 30% to 85% buffer B over 5 min and held constant for 5 min. Finally, the gradient was changed from 85% buffer B to 98% buffer A (100% water, 0.1% formic acid) over 1 min, and then held constant at 98% buffer A for 8 more minutes. The application of a 2.0 kV distal voltage electrosprayed the eluting peptides directly into the Orbitrap Fusion^TM^ Tribrid mass spectrometer equipped with an Easy-spray source (Thermo Finnigan, San Jose, CA). Full mass spectra was recorded on the peptides over a 400 to 1500 m/z range at 120,000 resolution, followed by tandem mass (MS/MS) CID (collision induced dissociation) events for a total of a 3sec cycle. Charge state dependent screening was turned off, and peptides with a charge state of 2–6 were analyzed. Mass spectrometer-scanning functions and HPLC gradients were controlled by the Xcalibur data system (Thermo Finnigan, San Jose, CA). Three technical replicates were run for each sample, and MS/MS data from technical replicates were merged for subsequent database search.

#### Database search and interpretation of MS/MS data

Tandem mass spectra from raw files were searched against a human protein database using the Proteome Discoverer 1.4 (Thermo Finnigan, San Jose, CA). The Proteome Discoverer application extracts relevant MS/MS spectra from the.raw file and determines the precursor charge state and the quality of the fragmentation spectrum. The Proteome Discoverer probability-based scoring system rates the relevance of the best matches found by the SEQUEST algorithm.[19] The human database was downloaded as FASTA-formatted sequences from Uniprot protein database (database released on June 18, 2014).[20] The peptide mass search tolerance was set to 10ppm. A minimum sequence length of 7 amino acids residues was required. Only fully tryptic peptides were considered. To calculate confidence levels and false discovery rates (FDR), Proteome Discoverer generates a decoy database containing reverse sequences of the non-decoy protein database and performs the search against this concatenated database (non-decoy + decoy).[21] The discriminant score was set at 5% FDR determined based on the number of accepted decoy database peptides to generate protein lists for this study. Spectral counts were used as the quantitative values for the protein-based list (distinct proteins). A unique peptide list was generated by including peptides assigned only to a single protein. Peak areas were used as the quantitative values for the unique-peptide list. The mass spectrometry dataset used to generate data in this study can be accessed via MassIVE database using study ID MSV000079352.

#### Statistical & bioinformatics analysis

Descriptive analyses are presented for the demographic/clinical characteristics and the percentage of participants in each cohort. Quantitative values from protein-based list or unique peptide-based list were normalized and analyzed by the Qlucore Omics Explorer (Qlucore AB, Sweden). Mann–Whitney–Wilcoxon test was applied to data to identify differentially expressed proteins between two groups of patients (with or without CIPN). Maximum false discovery rate (FDR) was controlled at 20% (q<0.2) and adjusted p-values were reported for statistical significance. An expanded list of proteins with 30% FDR (q<0.30) was used to perform Pathway/Network analyses using Ingenuity Pathway Analysis (IPA, Qiagen, CA) and MetaCore^TM^ (Thomson Reuters, NY).

## Results

### Patient demographic information

Baseline demographic and clinical characteristics are described in Table 1. The median age of the women enrolled was 49.5 years (SD 12.28). Age and body mass index (mean 28.8 kg/m^2^) were similar between the two groups. There were no current smokers in either group, and 66% had <1 alcoholic beverage per week. The taxane total dose and schedule and prior exposure to chemotherapy was also similar between the two groups. The group that developed CIPN was more likely to be white (6 vs. 3) and pre-menopausal (6 vs. 3); however, due to small sample size, formal tests for significance were not done.

**Table 1:** Baseline demographics and clinical characteristics by NTX subscale score.

Baseline Characteristic	Group 1: Increased Neuropathy (N = 8)			Group 2: No Change in Neuropathy (N = 9)			Total (N = 17)		
	No		%	No		%	No		%
Age at enrollment, years									
Mean		48.5			50.3			49.5	
SD		13.75			11.6			12.28	
Race									
White	6		75	3		33.33	9		52.94
Black or African American	2		25	6		67.67	8		47.06
Ethnicity									
Non-Hispanic	5		62.5	2		22.22	7		41.18
Hispanic	2		25	5		55.56	7		41.18
Other	1		12.5	2		22.22	3		17.65
Smoker									
Never smoked	3		37.5	2		22.22	5		29.41
Quit	5		62.5	7		77.78	12		70.59
Alcoholic beverages per week									
None	4		50	3		33.33	7		41.18
<1/week	2		25	2		22.22	4		23.53
1–6/week	1		12.5	3		33.33	4		23.53
Unknown	1		12.5	1		11.11	2		11.76
BMI									
Mean		29.38			28.22			28.76	
SD		10.16			2.99			7.07	
Menopause									
Pre-menopausal	6		75	3		33.33	9		52.94
Post-menopausal	2		25	6		67.67	8		47.06
Stage									
1	0		0	3		33.33	3		17.65
2	6		75	4		44.44	10		58.82
3	2		25	2		22.22	4		23.53
ER status									
Positive	6		75	5		55.56	11		64.71
Negative	2		25	4		44.44	6		35.29
PR status									
Positive	5		62.5	4		44.44	9		52.94
Negative	3		37.5	5		55.56	8		47.06
HER2 status									
Positive	1		12.5	1		11.11	2		11.76
Negative	7		87.5	8		88.89	15		88.24
Taxane regimen									
Tx4 q 2 wks	5		62.5	9		100	14		82.35
Tx6 q 2 wks	1		12.5	0		0	1		5.88
Tx12 q wk	2		25	0		0	2		11.76
Previous chemotherapy									
None	1		12.5	2		22.22	3		17.65
AC x 4 q 2 wks	6		75	7		77.78	13		76.47
AC x 6 q 3 wks	1		12.5	0		0	1		5.88

### MS-based workflow

**Fig 1:**
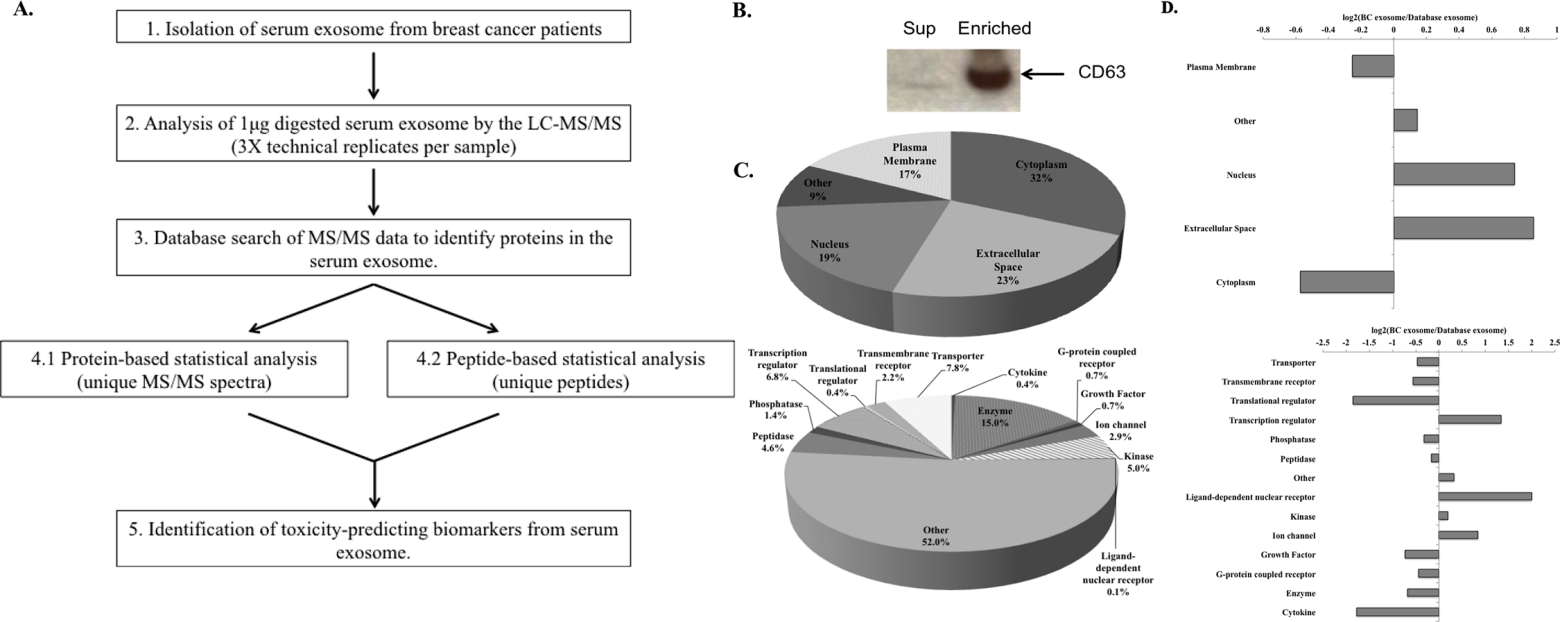
Isolation and characterization of serum exosomes derived from selected breast cancer patients. (A). Schematic illustration of the LC-MS/MS workflow used for biomarker discovery. (B). Western blot analysis of exosome enrichment by the presence of CD63. (C). Classification of serum exosome proteins by GeneOntology (GO) subcellular and molecular functions. (D). Comparison of GO classification between our dataset and the total exosome data from Vesiclepedia. Fold difference between our data and data from Vesiclepedia was plotted in a log scale on the x-axis.

We first optimized the method used to isolate total exosome from archived patient serum using the exosome isolation kit from Life Technologies Inc. (see Methods). Then, we analyzed proteins present in the serum exosome using the MS-based proteomics biomarker discovery workflow shown in Fig 1A. Enrichment of exosome from patient serum was shown by the detection of proteins enriched in exosomes such as CD63 in the exosome pellet and not in the supernatant (Fig 1B). A complex protein profile of serum exosome was generated from 5 μg of total exosome lysate using the highly sensitive Orbitrap Fusion Tribrid mass spectrometer. 879 proteins were identified from serum exosomes of selected breast cancer patients and 760 proteins were annotated by gene ontology annotation using the Ingenuity Pathway Analysis (IPA).

### Functional profiles of identified exosome proteome from serum of breast cancer patients

**Fig 2:**
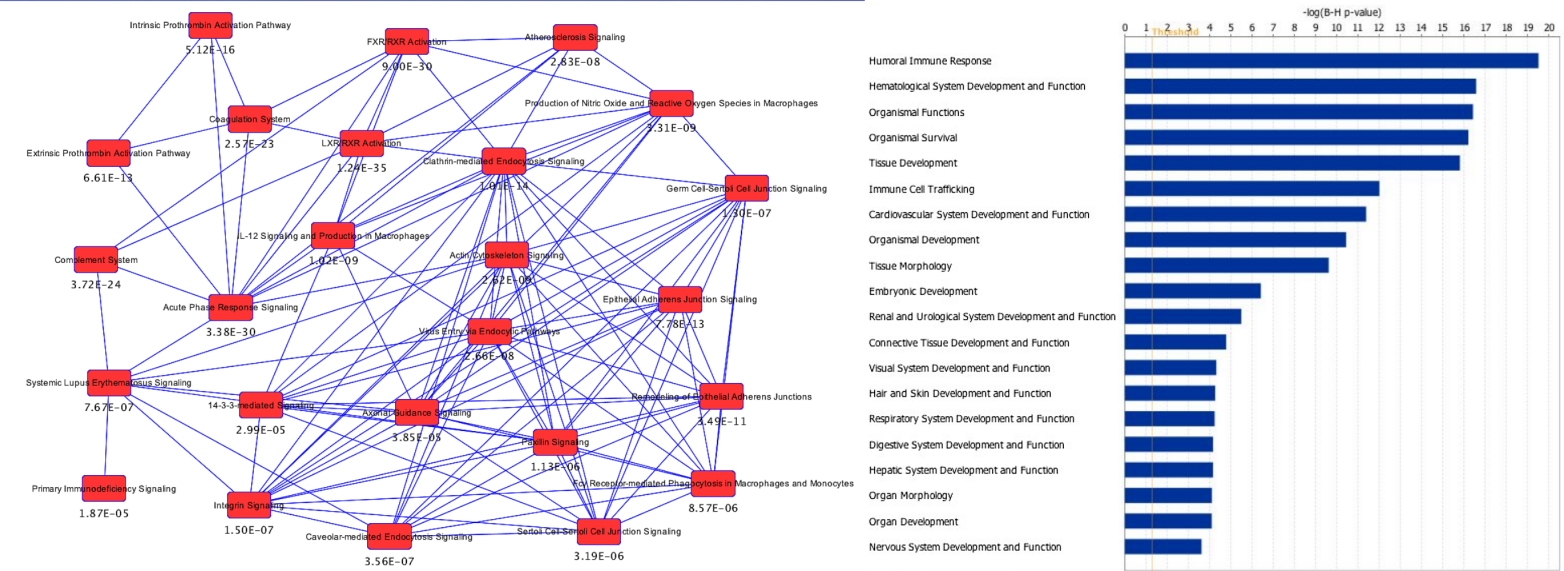
Pathway analysis of serum exosomes isolated from selected breast cancer patients derived from Ingenuity Pathway Analysis (IPA). (A). Overlapping canonical pathways that were significantly enriched in the dataset. B-H p-value was listed below each pathway. (B). Enriched pathways based on biological functions. Threshold indicates the minimal significance level [scored as–log (B-H p-value) from Benjamini-Hochberg procedure for multiple testing correction].

We identified a wide-range of proteins known to be in different subcellular locations (*e*.*g*., 32% cytoplasm and 19% nucleus) and have different functions (e.g. 15% enzymes, 6.8% transcription regulators, and 5% kinases) (Fig 1C). Our findings supported the concept that exosomes transport transcriptional and translational machinery that might have direct impact on the recipient cells. Then, we expanded our comparison to examine the subcellular and functional categories of proteins identified in our dataset to the proteomic data from the Vesiclepedia exosome database.[22] We found distinct differences in some of the categories between our data set and the Vesicleopedia database. For example, a substantial increase of nuclear and secreted proteins was observed in our dataset compared to the Vesicleopedia database (Fig 1D). In functional categories, we saw even more discrepancies. In our dataset, we observed a reduction of translational regulators and cytokines comparing to the Vesicleopedia database. On the other hand, we saw a significant increase of transcription regulator and ligand-dependent nuclear receptor in our data set comparing to the Vesicleopedia database (Fig 1D). Since tumors have been resected from these patients, these discrepancies could reflect their systemic response to anti-cancer treatments as opposed to collective protein profile in the Vesicleopedia database, which contain a large collection of exosome proteins secreted by cancer cells. Furthermore, IPA core analysis of these proteins revealed several immune response pathways were significantly enriched among other canonical pathways (Fig 2A), with the humoral immune response being the most significantly enriched molecular function (Fig 2B). It has been reported that exosome content could be dynamically regulated by the cellular microenvironment during pathological and normal conditions.[23–25] Together our findings support the idea that research into exosome cargo changes during disease may provide vital information to the understanding of cellular and systemic response to disease.

### Inclusion of peptide-based statistical analysis for ms-based biomarker discovery

**Fig 3:**
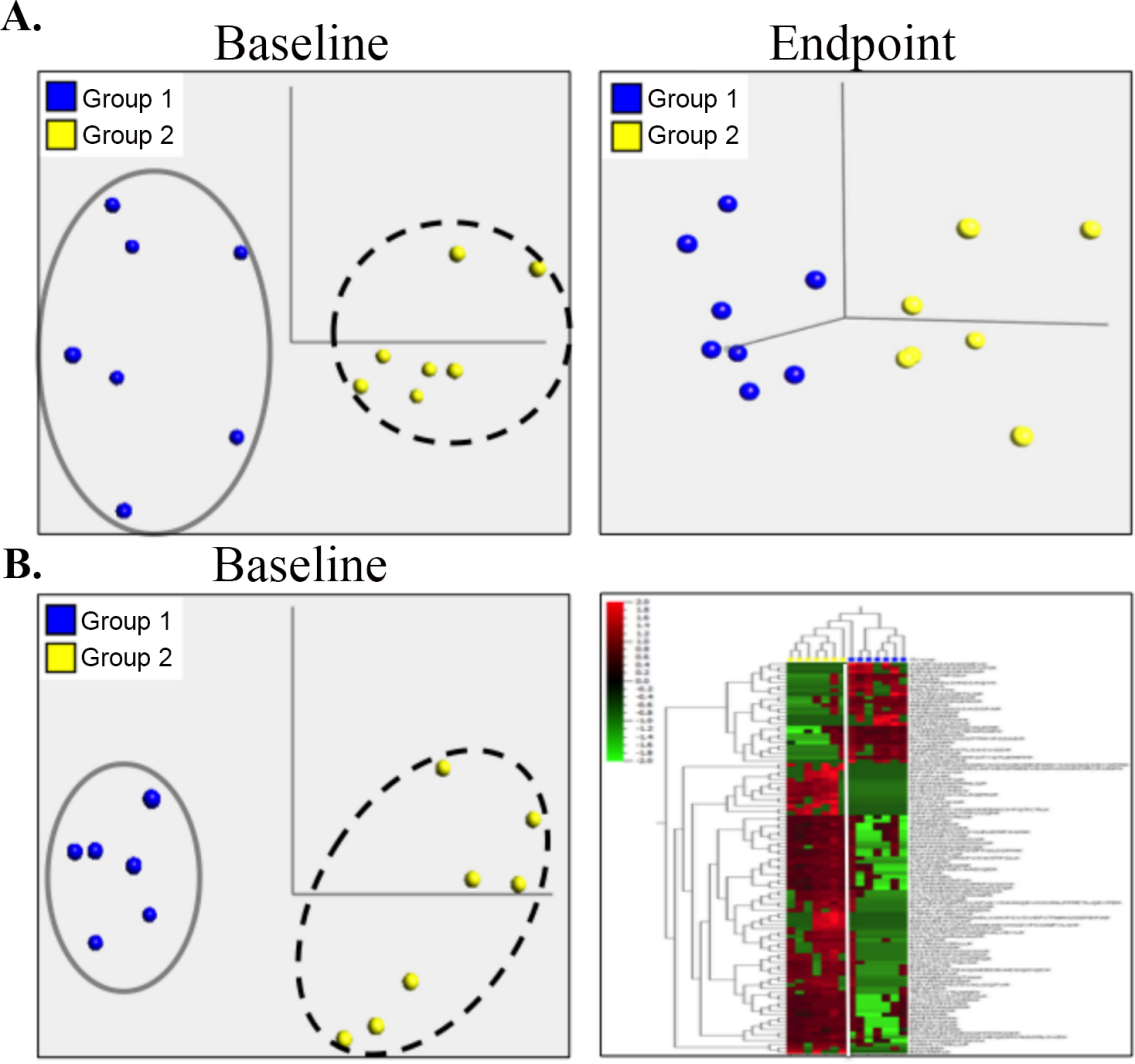
Statistical analysis of different protein lists using the Qlucore Omics Explorer. (A). Principal Component Analysis (PCA) of proteins identified in the serum exosome from baseline and endpoint blood draws for Group 1 (>20% increase in neuropathy) and Group 2 (no change in neuropathy). (B). PCA of proteins identified in the unique peptide-based list (left) and unsupervised hierarchical clustering of the unique peptide-based protein signature (right).

One challenge of quantitative assessment of MS-based proteomics data is peptide to protein assignment. Due to protein homology, a single peptide can be assigned to multiple proteins and counted multiple times in homologous proteins. This is also true for proteins with similar domains. To improve the quantification of MS-based approach for biomarker discovery, we designed a new strategy to compare and integrate protein- and peptide-based quantification. The protein-based approach uses unique spectra for quantification to reduce spectra duplication, and the unique peptide-based approach only includes peptides that are assigned to one protein and uses peak values (area under the curve) for quantification. By applying the Mann–Whitney–Wilcoxon test to both protein lists separately using the Qlucore Omics Explorer, we made two significant observations. First by applying the Principle Component Analysis (PCA) to the protein-based list with maximum 30% FDR, we observed a distinct separation of group 1 (>20% increase in neuropathy) and group 2 (no change in neuropathy) in serum exosome isolated from baseline blood draw (q<0.3, n = 11 proteins) (Fig 3A). On the contrary, the same statistical parameters yielded only 3 proteins that can distinguish group 1 from group 2 patients at the endpoint (12 month) blood draw suggesting that baseline blood draw is a better source for identifying toxicity predicting biomarkers than the endpoint blood draw. Secondly, applying the same statistical parameters to the peptide-based list yielded 36 proteins that can distinguish group 1 from group 2 patients at the baseline blood draw (Fig 3B). Among these 36 proteins, 10 proteins overlapped with the taxane toxicity-predicting biomarkers derived from the protein-based list. These findings suggest that unique peptide-based approach can be incorporated to increase the specificity and number of taxane toxicity-predicting biomarkers.

### Analysis of taxane toxicity-predicting biomarkers derived from serum exosome

Notably, 32 out of the 37-combined protein signature were found to express at higher levels in group 2 patients (no CIPN) compared to group 1 (CIPN) patients (S1 Table). With a further reduction of FDR to 20%, we identified a 12-protein protein signature, which contain potential candidates of predictive biomarkers for taxane-induced CIPN (S1 Table marked with *).

**Fig 4:**
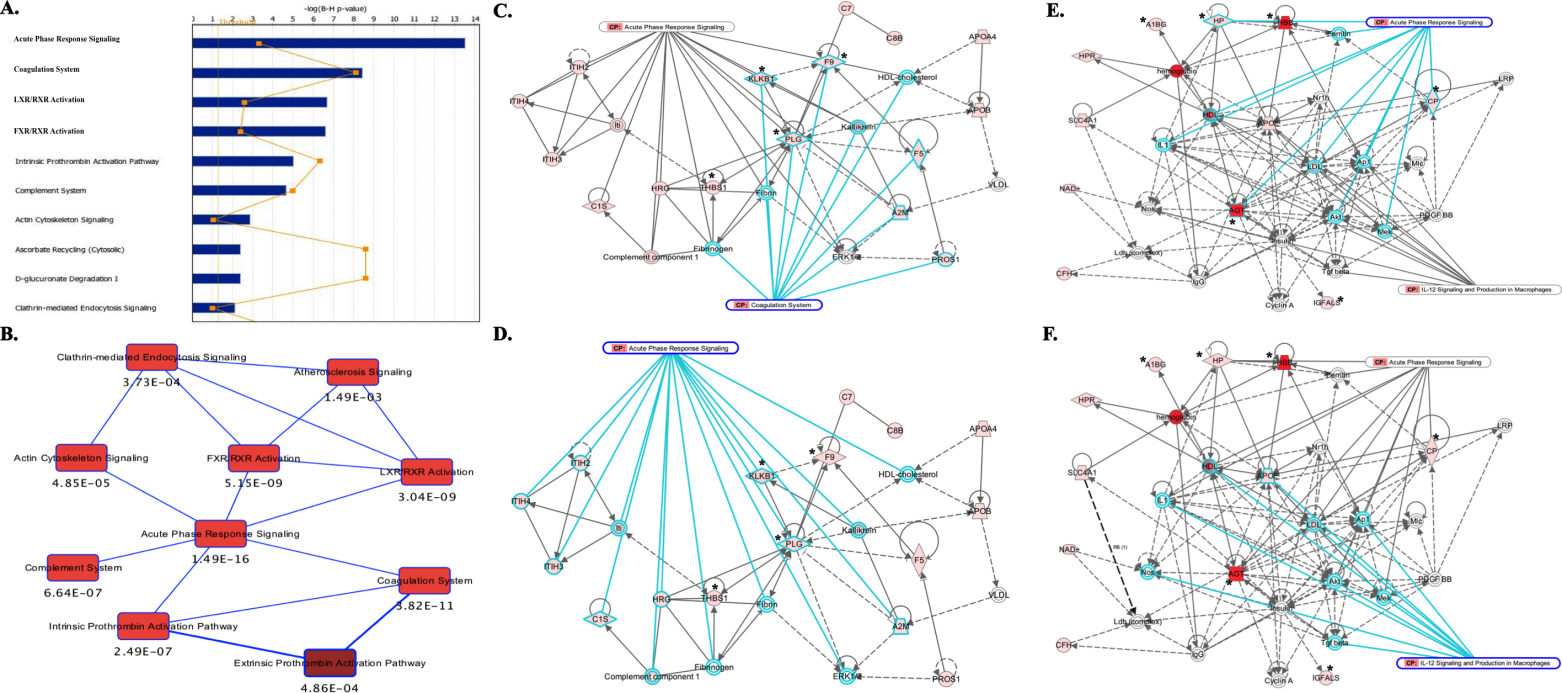
Pathway analysis of exosomal proteins expressed at higher levels in the group 2 breast cancer patients (taxane treated and no change in neuropathy) derived from Ingenuity Pathway Analysis (IPA). (A). Top 10 canonical pathways emerged after IPA core analysis. Threshold indicates the minimal significance level [scored as–log (B-H p-value) from Benjamini-Hochberg procedure for multiple testing correction]. (B). Overlapping canonical pathways that were significantly enriched in this dataset. B-H p-value was listed below each pathway. (C). Network #1 from significantly enriched canonical pathways displaying proteins involved in the network. Proteins identified in the network were colored in red. Blue lines connected interacting proteins in the Coagulation System. (D). Network #1 from significantly enriched canonical pathways displaying proteins involved in the network. Proteins identified in the network were colored in red. Blue lines connected interacting proteins in the Acute Phase Response Signaling. (E). Network #2 from significantly enriched canonical pathways displaying proteins involved in the network. Proteins identified in the network were colored in red. Blue lines connected interacting proteins in the Acute Phase Response Signaling. (F). Network #2 from significantly enriched canonical pathways displaying proteins involved in the network. Proteins identified in the network were colored in red. Blue lines connected interacting proteins in the IL-12 signaling and production in macrophages. Significantly different proteins (q<0.2) were marked by *.

**Fig 5:**
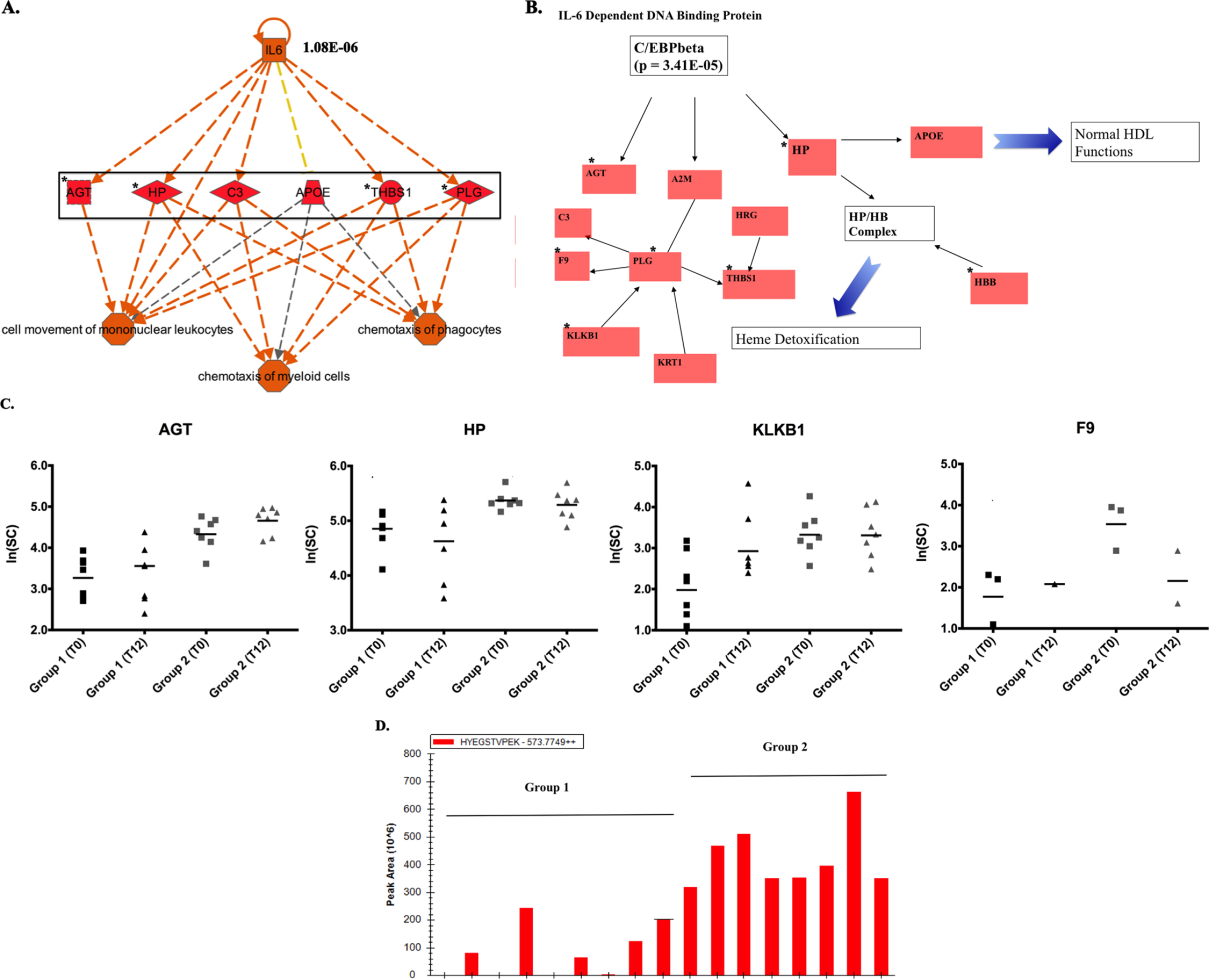
Pro-inflammatory signaling was identified in the exosomal proteins expressed at higher levels in the group 2 breast cancer patients (taxane treated and no change in neuropathy) based on the upstream analysis of IPA. (A). Higher levels of IL-6 targets found in the group 2 breast cancer patients compared to the group 1 breast cancer patients. P-value of the prediction is listed next to the upstream regulator, IL-6. (B). Interacting networks of C/EBPβ signaling proteins found in the group 2 breast cancer patients and their predicted physiological functions. (C). Scatter plots of representative proteins in the pro-inflammatory signaling networks in group 1 and 2 breast cancer patients and their expression in two time points (T0 and T12). T0 represents the baseline blood draws, and T12 represents the endpoint blood draws. Spectral counts of these proteins in patients were represented in the linear log scale (ln). p-values are included in the comparison between the group 1 and group 2 patients at the baseline. (D). Peak area of a peptide, HYEGSTVPEK (2+), from haptoglobin (HP) showed higher level of expression in group 2 patients than in group 1 patients. Significantly different proteins (q<0.2) were marked by *.

IPA core analysis of the 37-protein signature revealed that immune responses were among the top significantly enriched canonical pathways (Fig 4A). Two of the top 10 canonical pathways are acute phase response (p-value 1.49E-16) and coagulation system (p-value 3.82E-11) (Fig 4B). The top two networks that connect the most proteins identified in the dataset also suggested an enrichment of immune response related signaling. The top two interactomes that connect network 1 (20 focus molecules) are coagulation system (Fig 4C) and acute phase response signaling (Fig 4D). Plasminogen (PLG) seems to be the key protein that links these two pathways in network 1. The top two interactomes that connect network 2 (11 focus molecules) are acute phase signaling (Fig 4E) and IL-12 signaling and production in macrophages (Fig 4F). LDL complex seems to be the key in linking these two pathways in network 2. To identify upstream transcriptional regulators that can explain the observed protein changes in our dataset, we utilized IPA upstream regulator analysis and found two transcriptional regulators, IL6 (p-value 1.08E-06) and C/EBPβ (p-value 3.41E-05) that are predicted to be the key regulators of proteins elevated in patients without CIPN after taxane treatment based on the activation of their downstream targets (Fig 5A and 5B). Examples of relative differences of identified targets between group 1 and group 2 patients as well as differences between two time points within the patient group (T0 = baseline prior to taxane treatment; T12 = endpoint) were shown to further support the findings from pathway-based analyses (Fig 5C). Log values of unique spectral counts were used as semi-quantitative measurements for statistical analysis of expression trends. An example of one biomarker, Haptoglobin (HP) expression using peak-based quantification is shown in Fig 5D. Together, our findings strongly indicate that patients showing no evidence of taxane-induced CIPN had elevated inflammatory and detoxification responses after the primary chemotherapy and these responses persisted after receiving taxane as adjuvant treatment.

## Discussion

In summary, we have demonstrated the feasibility of identifying biomarkers from serum exosomes of archived samples from breast cancer patients. We have also identified a novel panel of biomarkers from serum exosomes that were associated with the development of taxane-induced peripheral neuropathy. Importantly, our data suggests that patients who had depressed or low inflammatory and detoxification responses prior to taxane treatment are at increased risk to develop taxane-induced peripheral neuropathy. We believe that this new panel of biomarkers could be used to identify patients at high risk of developing severe CIPN.

Prior research in this area has focused on germline genetic variability (e.g., SNPs, copy number variations, etc.) to predict drug-induced toxicity. Germline variants can impact the metabolism, transport, and excretion of drugs, but can also impact the target tissue. A candidate study from a large breast cancer trial demonstrated an association between a SNP in *FANCD2* and taxane induced neuropathy.[8] However another large trial identified a SNP in *FDG4* that correlated with increased likelihood of paclitaxel-induced peripheral neuropathy.[9] *FDG4* is associated with the hereditary neuropathy condition of Charcot-Marie-Tooth disease. Finally, a study specifically focused on more rare variants using massively parallel sequencing of 20,794 genes associated with heredity neuropathy from patients who had received paclitaxel-based chemotherapy,[26] reported an association between *EPHA5*, *ARHGEF10*, *and PRX* and paclitaxel-induced peripheral neuropathy. Concerns have been raised about the lack of consistency from one study to the next. Many of these studies suffered from incomplete characterization of the phenotype of CIPN.

Studies using MS-based high-throughput techniques to facilitate biomarker discovery have been reported.[27–31] Serum, which can be obtained routinely from patients for continuous monitoring of treatment efficacy, are ideal sources to identify early, predictive, and minimally invasive therapy-induced toxicity biomarkers. Secreted extracellular vesicles such as exosomes have been reported to remain intact in biofluids during long-term storage, and therefore serve as an excellent reservoir for biomarker discovery. It has become increasingly clear that exosomes have specialized functions.[16] Proteomic cataloguing of exosomes from diverse cell types has revealed a common set of membrane and cytosolic proteins, suggesting the evolutionary importance of these membrane particles.[16, 17] These studies also demonstrated that when exosomes are actively secreted by live cells, they represent a new type of intercellular messenger. Findings from our studies further support the utility of using serum exosomes for MS-based biomarker discovery.

Currently, the exact mechanism of taxane-induced peripheral neuropathy is not well understood. We found baseline inflammatory state is associated with the protection of CIPN, in that patients with elevation of proteins regulated by the two transcriptional regulators, IL-6 and C/EBPβ, were less likely to develop the toxicity. IL-6 is a pro-inflammatory cytokine that leads to systemic activation of inflammatory immune response.[32] C/EBPβ is an important transcription factor regulating the expression of genes involved in immune and inflammatory responses.[33, 34] It binds to regulatory regions of several acute-phase and cytokines genes (*e*.*g*. IL-6) and probably plays a role in the regulation of acute-phase reaction, inflammation and hematopoiesis.[34, 35] It’s downstream target, haptoglobin (HP) is important in maintaining normal HDL functions as well as heme detoxification.

Research on the interaction between inflammation and neuropathic pain has resulted in conflicting results. For instance, upregulation of chemokines, which are responsible for coordinating the response of immune cells, were observed in animal models of CIPN.[36, 37] Other studies measured levels of inflammatory markers in the presence of neuropathy. Most of these studies evaluated the inflammatory markers after the subjects became symptomatic and the results were conflicting.[38, 39] On the other hand, in a mouse model, Flatters *et al*. found that after nerve injury, peripheral exogenous administration of IL-6 produced inhibition of spinal neuronal responses to noxious mechanical stimulation.[40] This finding supports the protective effect of IL-6 as extrapolated in our study. Although the scope of this study was to find predictive biomarkers for CIPN, future studies will focus on response to treatment and will investigate changes of exosome proteins over time. This line of investigation will provide new insights on the mechanism of CIPN.

We also believe the collective protein signature we found may serve as better predictors than a single biomarker for taxane-induced neuropathy to identify patients at high risk of developing severe CIPN. The association between each of the proteins and CIPN on this panel might not be immediately apparent when examining them individually, however there are hypotheses about the relationships that should be examined further. For example, Hemoglobin subunit beta (HBB) is highly expressed in erythrocytes and functions as an oxygen transporter by binding to hemoglobin. It can play a role in blood coagulation, regulation of blood pressure and other related biological processes. HBB has been found in exosomes derived from several cell types. For example, HBB was found in exosomes from viral infected B-cell [41] and endothelial cells in response to cellular injury.[42] Furthermore, it has been reported that hemoglobin chains are expressed in mammalian brain neurons and are regulated by treatments that affect mitochondria suggesting a novel role in neuronal response to injury.[43]

Another protein on this panel, angiotensinogen (AGT), is an essential component of the renin-angiotensin system (RAS). AGT has been identified in exosomes derived from neutrophils isolated from vascular and inflammatory diseases[44] and exosomes derived from human bone marrow mesenchymal stem cells found to promote proliferation and confer resistance of tubular epithelial cells to apoptosis.[45] More importantly when we examined interactions among these proteins collectively as shown in Figs 4 and 5, we found a prominent theme of homeostatic regulation. Our results suggest that taxane-treated patients who do not develop severe neuropathy may restore the homeostatic control. On the other hand, taxane-treated patients who did develop severe neuropathy did not regain homeostatic control. The exosomal signature we found in our patient cohort pointes to the response of the homeostatic balance between inflammation and detoxification. Our results are hypothesis generating and prompt us to ask new questions. For example, do taxanes contribute directly to neuropathy or are neurons more susceptible to systemic cytotoxic response when patients are treated with taxanes? Since we found more distinct exosomal proteins at baseline between these two groups of patients we speculate that there are host-factors that could predispose patients to this toxicity.

There are several strengths in this study. Our study is one of the few longitudinal studies, which use the sample from the same individual immediately before treatment as the baseline reference for progressive changes. Many studies have used “healthy individuals” as the baseline for changes that occurred in disease patients. The major problems for these types of studies are i) heterogeneous genetic background, and ii) unknown underlying conditions in so-called healthy individuals. Additionally, we used the FACT-Ntx score as the primary outcome measure of the cohort study, which is a validated patient reported outcome, and this measure provides a more sensitive and accurate assessment of neuropathy as compared to the CTCAE. Our study also has limitations. The small sample size of 17 patients decreases the robustness of our findings. In addition, we did not have a separate validation set. We plan to validate the 12-protein biomarker signature in a larger cohort of 409 patients who have received adjuvant taxane therapy for breast cancer.[46] We also recognized that there were still major plasma proteins present in the CD63 enriched total exosome preparation. However, other groups had similar findings that current exosome isolation methods could reduce the presence but not totally exclude plasma proteins.[47] In the future, extensive purification of exosomes (e.g. differential centrifugation or immuno-affinity purifications) will allow us to dig deeper into the lower abundant but pathologically relevant proteins present in the serum exosomes.

In summary, we have identified a panel of 12 protein signature associated with the development of taxane-induced peripheral neuropathy. We have also uncovered a potential mechanism that may be associated with the development of this toxicity. Understanding the mechanism for CIPN is critical toward identifying potential targets for preventive and therapeutic interventions. Future studies will focus on validating the biomarker panel in a larger cohort. Understanding factors that lead to increased toxicity will allow for more personalized therapy in order to optimize treatment efficacy and improve long-term quality of life in cancer survivors.

## Supporting Information

S1 Table 37-combined protein signature (q<0.3) and 12 candidate taxane toxicity predictive protein markers. (DOCX)
