# Supplementary material for: Identifying Predictors of Taxane-Induced Peripheral Neuropathy Using Mass Spectrometry-Based Proteomics Technology
Source: PLoS One. 2015 Dec 28;10(12):e0145816. doi: 10.1371/journal.pone.0145816 (PMC4692419; doi:10.1371/journal.pone.0145816)
Supplement: S1 Table — (DOCX) [file pone.0145816.s001.docx]

**S1 Table: 37-combined protein signature (q<0.3) and 12 candidate taxane toxicity predictive protein markers.**

a. Top panel is protein-based protein list and bottom panel is peptide-based protein list.

b. * markes proteins with q<0.2
